# The acute phase response and soman-induced status epilepticus: temporal, regional and cellular changes in rat brain cytokine concentrations

**DOI:** 10.1186/1742-2094-7-40

**Published:** 2010-07-22

**Authors:** Erik A Johnson, Robert K Kan

**Affiliations:** 1 Research Division, Pharmacology Branch, US Army Medical Research Institute of Chemical Defense (USAMRICD), Aberdeen Proving Ground, MD 21010, USA

## Abstract

**Background:**

Neuroinflammation occurs following brain injury, including soman (GD) induced status epilepticus (SE), and may contribute to loss of neural tissue and declined behavioral function. However, little is known about this important pathological process following GD exposure. Limited transcriptional information on a small number of brain-expressed inflammatory mediators has been shown following GD-induced SE and even less information on protein upregulation has been elucidated. The purpose of this study is to further characterize the regional and temporal progression of the neuroinflammatory process following acute GD-induced SE.

**Methods:**

The protein levels of 10 cytokines was quantified using bead multiplex immunoassays in damaged brain regions (i.e., piriform cortex, hippocampus and thalamus) up to 72 hours following seizure onset. Those factors showing significant changes were then localized to neural cells using fluorescent IHC.

**Results:**

A significant concentration increase was observed in all injured brain regions for four acute phase response (APR) induction cytokines: interleukin (IL)-1α, IL-1β, IL-6, and tumor necrosis factor (TNF)-α. Increases in these APR cytokines corresponded both temporally and regionally to areas of known seizure damage and neuronal death. Neurotoxic cytokines IL-1α and IL-1β were primarily expressed by activated microglia whereas the potentially neuroprotective cytokine IL-6 was expressed by neurons and hypertrophic astrocytes.

**Conclusions:**

Increases in neurotoxic cytokines likely play an active role in the progression of GD-induced SE neuropathology though the exact role that these and other cytokines play in this process require further study.

## Background

Chemical warfare nerve agents (CWNA) were developed during World War II but remain a significant threat through deployment by hostile nations or by terrorist organizations [1]. CWNA, such as soman (pinacolyl methylphosphonofluoridate, GD), rapidly and irreversibly bind to acetylcholinesterase, causing excess acetylcholine accumulation in the central and peripheral nervous systems. GD exposure can cause intense tonic-clonic convulsions, respiratory paralysis and possibly death [2]. Following exposure, the ensuing cholinergic crisis leads to the development of status epilepticus (SE) that can continue unabated for many hours [3]. SE induces neuroinflammatory gliosis [4] and profound neuronal cell loss in the piriform cortex, hippocampus, amygdala and thalamus [5,6]. Excitotoxic neural damage following GD exposure activates a neuroinflammatory response [7-10], which may contribute to the neuropathology.

The extent to which neuroinflammation contributes to cell loss following central nervous system (CNS) injury largely depends on many factors, such as local environment, concentration of the inflammatory mediators, the responding immune cell phenotype and the timing of their interaction with damaged neural cells [11,12]. In severe and progressive CNS injuries, increased neuroinflammatory activity appears detrimental since anti-inflammatory treatments are successful in reducing brain pathology in animal models of CNS injury [13,14]. Following injury, infiltrating leukocytes and activated macrophages release many inflammatory proteins, including the acute phase response (APR) inducing cytokines IL-1, IL-6 and TNF-α [15]. Though pluripotent, cytokines such as IL-1 (α and β) and TNF-α are toxic to neural tissues *in vitro *[16-18] and can exacerbate experimental CNS injury *in vivo *[19-21].

Evidence of neuroinflammation following GD-induced SE has been shown at the level of gene transcription [7-10], though data are limited on protein upregulation [22,23]. Therefore, the purpose of this study was to investigate the extent and maturation of the neuroinflammatory response by examining cytokine protein increases following GD exposure up to 72 hours after SE onset. Protein levels of ten cytokines were quantified using a multiplex bead immunoassay in brain tissue lysates of SE-injury susceptible regions (i.e., piriform cortex, thalamus and hippocampus). APR cytokines were markedly elevated in vulnerable brain regions and were localized to resident neural cells (i.e., neurons, astrocytes or microglia). These data are the first to show concurrent cytokine protein upregulation and cellular origin of these factors following GD-induced SE.

## Methods

### Animals

Adult male Sprague-Dawley rats (Charles River Laboratories, Wilmington, MA; CRL: CD[SD]-BR, 250 - 350 g) were treated with HI-6 dichloride (Walter Reed Army Institute of Research, Silver Spring, MD; 125 mg/kg, i.p.) 30 minutes prior to GD administration and with atropine methyl nitrate (AMN, Sigma-Aldrich, St. Louis, MO; 2.0 mg/kg, i.m.) 1 minute after GD administration. Vehicle control animals received HI-6, AMN and saline, while naïve animals received no injections. GD (GD-U-2323-CTF-N, purity 98.8 wt%) was diluted in saline at USAMRICD. GD (1.6 LD_50 _= 180 μg/kg) was administered subcutaneously in the scruff of the neck and the rat was observed for convulsive activity. This dose of GD produces within minutes [24] a 100% generalized convulsive seizure rate that is maintained up to 24 hours [3]. The experimental protocol was approved by the Animal Care and Use Committee at the United States Army Medical Research Institute of Chemical Defense and all procedures were conducted in accordance with the principles stated in the Guide for the Care and Use of Laboratory Animals (National Research Council, 1996), and the Animal Welfare Act of 1966 (P.L. 89-544), as amended. The animal care program at this institute is fully accredited by the Association for Assessment and Accreditation of Laboratory Animal Care International.

### Multiplex bead array immunoassay

Experimental, vehicle control and naïve animals were deeply anesthetized with a sodium pentobarbital solution (70 mg/kg, i.p.) then euthanized by decapitation at 0.5, 1, 3, 6, 12, 24, 48 or 72 hours after onset of convulsions. Following euthanasia, piriform cortex, hippocampus and thalamus tissue was extracted and processed into lysate as previously described [25]. Briefly, the brain regions were excised, rinsed with cold PBS and snap frozen in liquid nitrogen. The tissues were weighed and homogenized in ice-cold triple detergent lysis buffer containing a Complete™ protease inhibitor cocktail (Roche Biochemicals, Indianapolis, IN) at a ratio of 1 ml buffer to 50 mg tissue. Samples were allowed to stand at 4°C for at least 30 minutes before centrifugation at 8000 G for 5 minutes and removal of the lysate for assaying. Cytokine concentrations were quantified using a rat cytokine multiplex bead immunoassay kit containing IL-1α, IL-1β, IL-2, IL-4, IL-6, IL-10, IL-12p70, IL-13, IL-17, and TNF-α (LINCO Research, St. Charles, MO). The bead immunoassay procedure used 25 μl of sample (94 ± 8 μg protein) per well and was conducted according to the manufacturer's instructions with each individual cytokine standard curve and sample assayed in duplicate. The plate was read on a Luminex™ 100 instrument (Bio-Rad Laboratories, Hercules, CA) and analyzed with either BioRad or STaRStation software (Applied Cytometry, Sacramento, CA). The number of replicates for the experimental samples are as follows: piriform cortex, n = 6 for each time point and naïve; hippocampus, n = 6 for each time point except for naïve (n = 5), 6 hr (n = 5) and 24 hr (n = 7); thalamus, n = 5 for each time point and naïve except for 0.5 hr (n = 6), 6 hr (n = 4), 12 hr (n = 3), 24 hr (n = 6) and 48 hr (n = 6). Time matched vehicle controls (n = 3 per time point) were analyzed individually and condensed into a single vehicle control comparison group when no significant difference was found between these samples over time by analyte or brain region.

### Immunohistochemistry (IHC)

Separate from the animals used in the multiplex bead array immunoassay, experimental, vehicle control and naïve animals were deeply anesthetized, euthanized by decapitation at 12 hours after seizure onset and perfused with isotonic saline followed by 4% paraformaldehyde via cardiac puncture. Brains were immediately frozen at -20°C, cut on a Leica CM3050 S cryostat (Thermo Shandon, Inc.; Pittsburgh, PA) at 40 microns and stored in cryobuffer (30% each of glycerol, ethylene glycol and water, 10% 2 × phosphate buffer) until use. Free float fluorescent IHC labeling was conducted as previously described [25]. Experimental and vehicle control samples had an n = 3 for each cytokine/cell type combination. The antibodies used were as follows: rabbit anti-IL-1α (1:1000; ab9875, Abcam, Cambridge, MA), rabbit anti-IL-1β (1:1000; ab9787, Abcam), rabbit anti-IL-6 (1:500; ab6672, Abcam), mouse anti-NeuN to label neurons (1:1000; MAB377, Chemicon, Temecula, CA), mouse anti-GFAP to label astrocytes (1:1000; MS-280-P, NeoMarkers, Fremont, CA), and mouse anti-cd11b to label microglia and macrophages (1:1000; CBL1512, Chemicon). Alexafluor™ fluorescent-tagged secondary and tertiary antibodies (Molecular Probes, Eugene, OR) were used for visualization. Tissue sections labeled with only secondary and tertiary antibodies were used as secondary controls. Sections were viewed and digitally captured with an Olympus BX51 microscope equipped with an Olympus DP-70 high-resolution color CCD digital camera (Opelco, Dulles, VA). An Olympus BX61 equipped with a DSU spinning disk confocal system and DP-70 CCD camera was used to confirm same cell co-localization (Opelco). Images of 40 μm tissues were acquired using a z step interval of 1 μm and analyzed using Slidebook™ software (Opelco). Publication images were compiled using Adobe Photoshop CS digital image software. Color levels and background labeling were reduced and evened using the levels tool. All input levels (0-255) were normalized in the RGB channel as follows: highlight input levels were set at the peak of the image histogram, midtone levels were set at 0.8 and shadow levels were set either at the edge of the histogram closest to 255 or at 180, whichever was greater.

### Statistical analysis

Immunoassay data were evaluated by ANOVA with a post-hoc Dunnett's analysis and expressed in pg/ml. Data points calculated below the minimum detectible concentrations (MinDC) for the assays were conservatively set at -0.01 pg/ml of the MinDC for statistical analyses. Values are expressed as mean ± SEM. Differences were considered significant at the level of p ≤ 0.05.

## Results

### APR cytokines significantly increase following GD induced SE

**Figure 1:**
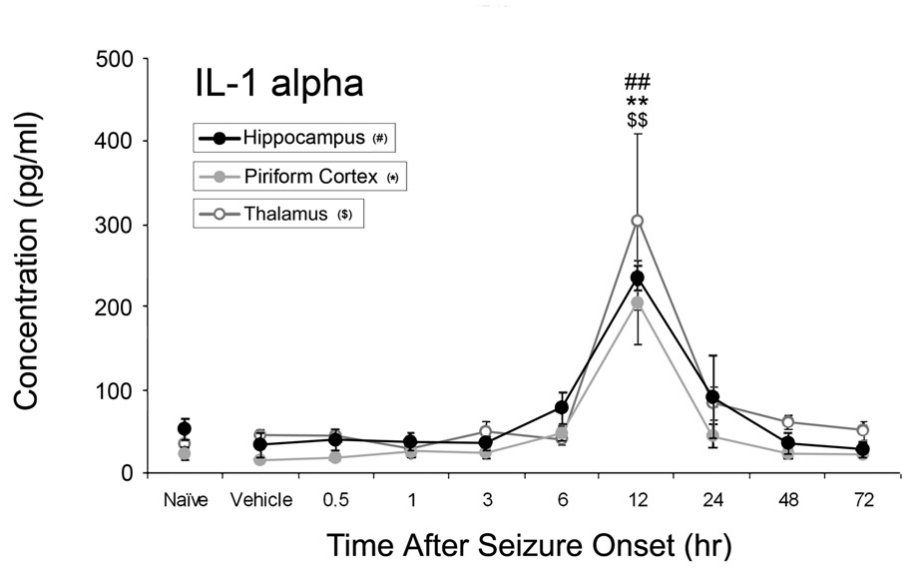
**IL-1α significantly increases in rat brain after GD-induced SE**. IL-1α concentrations significantly increase in the hippocampus, piriform cortex and thalamus 12 hours following GD-induced seizure activity. Concentrations of IL-1α peak at 12 hours in the piriform cortex (solid gray line), hippocampus (solid black line) and thalamus (open gray line). ^## ^p < 0.01 versus vehicle control in hippocampus, ** p < 0.01 versus vehicle control in piriform cortex, ^$$ ^p < 0.01 versus vehicle control in thalamus.

**Figure 2:**
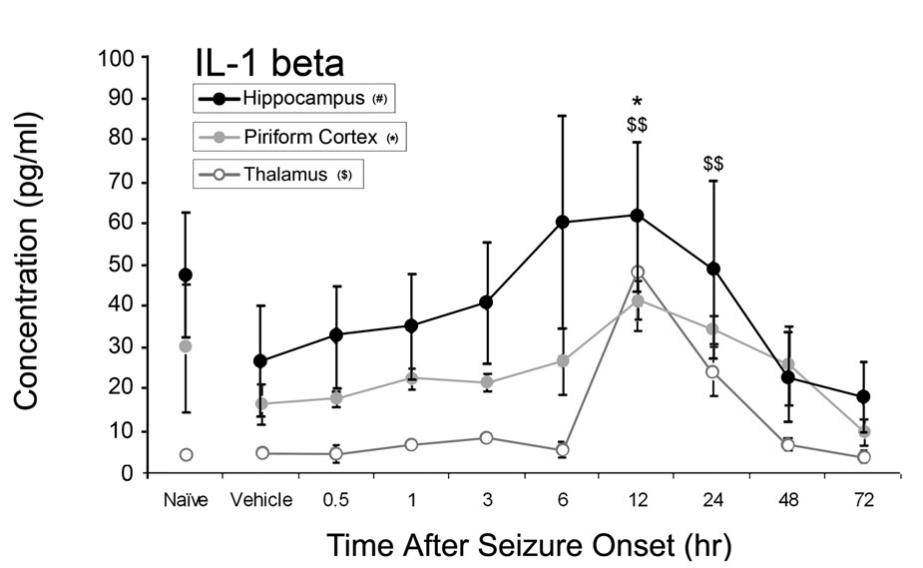
**IL-1β significantly increases in rat brain after GD-induced SE**. Though much less robust than IL-1α, IL-1β concentrations significantly increase in the piriform cortex twelve hours following GD-induced seizure activity (solid gray line). Concentrations of IL-1β also significantly increase in the thalamus (open gray line) though not in the hippocampus (solid black line). Also unlike IL-1α, IL-1β concentrations in naives were high and variable compared to experimental data. * p < 0.05 versus vehicle control in piriform cortex, ^$$ ^p < 0.01 versus vehicle control in thalamus.

**Figure 3:**
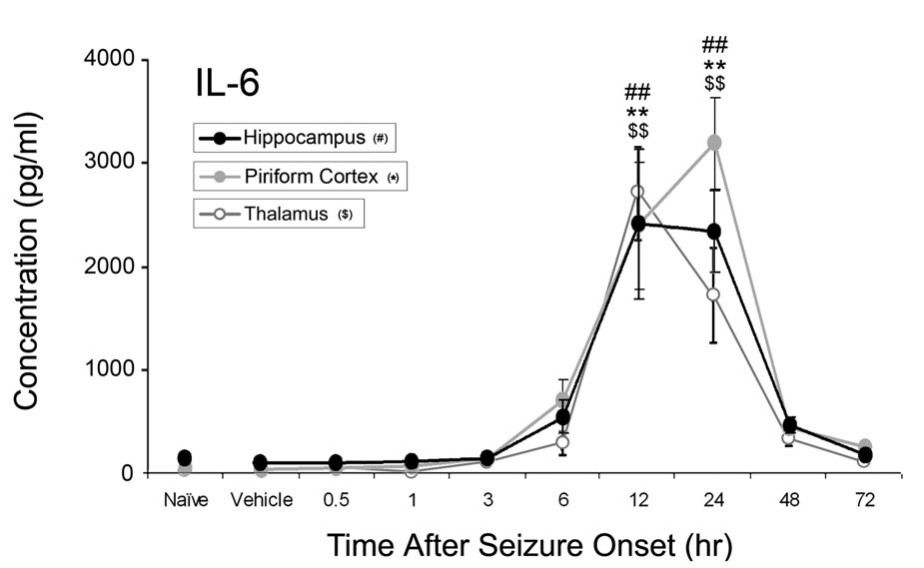
**IL-6 significantly increases in rat brain after GD-induced SE**. IL-6 concentrations significantly increase in the hippocampus, piriform cortex and thalamus 12 and 24 hours following GD-induced seizure activity. Concentrations of IL-6 peak at 24 hours in the piriform cortex (solid gray line), and at 12 hours in the hippocampus (solid black line) and thalamus (open gray line). ^## ^p < 0.01 versus vehicle control in hippocampus, ** p < 0.01 versus vehicle control in piriform cortex, ^$$ ^p < 0.01 versus vehicle control in thalamus.

**Figure 4:**
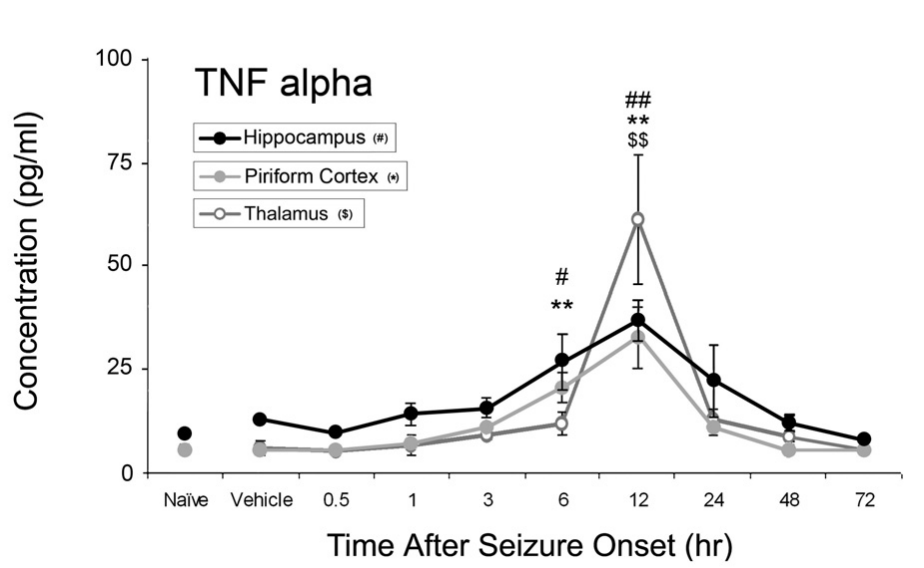
**TNF-α significantly increases in rat brain after GD-induced SE**. Concentrations of TNF-α peak at 12 hours in the piriform cortex (solid gray line), hippocampus (solid black line) and thalamus (open gray line). Significant increases were also seen in the hippocampus and piriform cortex at 6 hours following GD-induced SE. ^# ^p < 0.05, ^## ^p < 0.01 versus vehicle control in hippocampus, ** p < 0.01 versus vehicle control in piriform cortex, ^$$ ^p < 0.01 versus vehicle control in thalamus.

Of the ten inflammatory cytokines investigated, concentrations of four significantly increased: IL-1α, IL-1β, IL-6, and TNF-α as shown in Figures 1-4. No significant changes were observed for IL-2, IL-4, IL-10, IL-12p70, IL-13 or IL-17 during the time course (data not shown). Concentrations of IL-1α significantly increased at 12 hours after SE onset in the piriform cortex (205 ± 99 pg/ml v. 14 ± 7 pg/ml vehicle control), hippocampus (234 ± 29 pg/ml v. 34 ± 29 pg/ml vehicle control) and thalamus (303 ± 213 pg/ml v. 45 ± 13 pg/ml vehicle control)(Figure 1). IL-1β significantly increased in the piriform cortex at 12 hours (41 ± 10 pg/ml v. 16 ± 10 pg/ml vehicle control) and in the thalamus at 12 hours (48 ± 28 pg/ml) and 24 hours (24 ± 12 pg/ml v. 5 ± 1 pg/ml vehicle control) (Figure 2). No significant changes in IL-1β concentration were observed in the hippocampus though a definite trend was observed, with an approximate 3-fold increase over vehicle control values. Significant IL-6 concentration increases were observed in all brain regions at 12 and 24 hours (Figure 3) though peak expression varied by region. In the piriform cortex, IL-6 concentrations significantly increased at 12 hours (2399 ± 1238 pg/ml) and peaked at 24 hours (3200 ± 894 pg/ml) compared to vehicle control (38 ± 8 pg/ml). Peak levels in the hippocampus were observed at 12 hours (2462 ± 1489 pg/ml) with a small decrease at 24 hours (2386 ± 814 pg/ml v. 99 ± 48 pg/ml vehicle control) with the true peak likely occurring between these time points. In the thalamus, IL-6 peaks at 12 hours (2706 ± 913 pg/ml) though concentration increases are still significant at 24 hours (1711 ± 909 pg/ml) compared to vehicle control (13 ± 13 pg/ml). Lastly, TNF-α significantly increased in the piriform cortex at 6 (20 ± 7 pg/ml) and 12 hours (32 ± 15 pg/ml v. 4.43 ± 0.0 pg/ml [below MinDC]), in the hippocampus at 6 (27 ± 7 pg/ml) and 12 hours (37 ± 10 pg/ml v. 13 ± 3 pg/ml vehicle control) and in the thalamus at 12 hours (61 ± 31 pg/ml v. 6 ± 3 pg/ml vehicle control) (Figure 4). Naïve and vehicle controls were not significantly different from each other for any factor measured in any region. To further identify the source of these cytokines, neurons, astrocytes and microglia expressing IL-1α, IL-1β and IL-6 were identified in each brain region using IHC at the 12-hour time point, the peak of cytokine expression for the majority of these brain regions. Localization of TNF-α was not pursued due to a lack of effective IHC antibodies.

### IL-1 is expressed by microglia following GD induced SE

**Figure 5:**
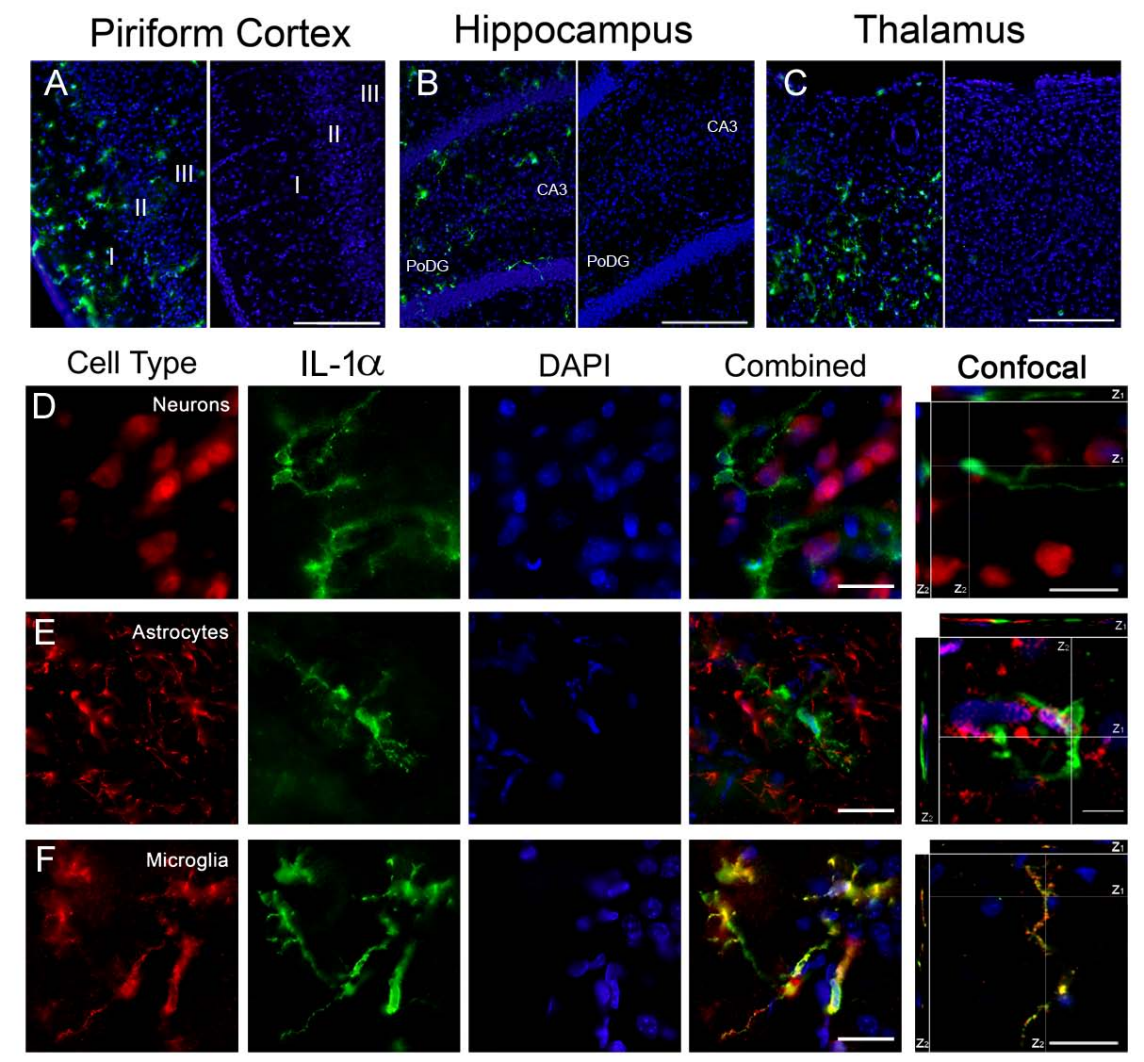
**IL-1α is expressed in activated microglia following GD-induced SE**. IL-1α (green) is present in the piriform cortex, hippocampus and thalamus 12 hours after GD-induced SE (A, B & C left), though absent in vehicle controls (A, B & C right). Neurons (D, red) and astrocytes (E, red) do not express IL-1α. Co-localization was observed in activated microglia (F, red). DAPI (A-F, blue) labels nuclei. For confocal images, the white lines indicate the vertical (Z) sectioning of the image and are shown as Z_1 _and Z_2 _on the edges of the main image. (Scale bar: 250 μm (A-C), 50 μm and 10 μm (D-F) for regular and confocal fluorescent microscopy respectively; n = 3 for each cytokine/cell type combination)

**Figure 6:**
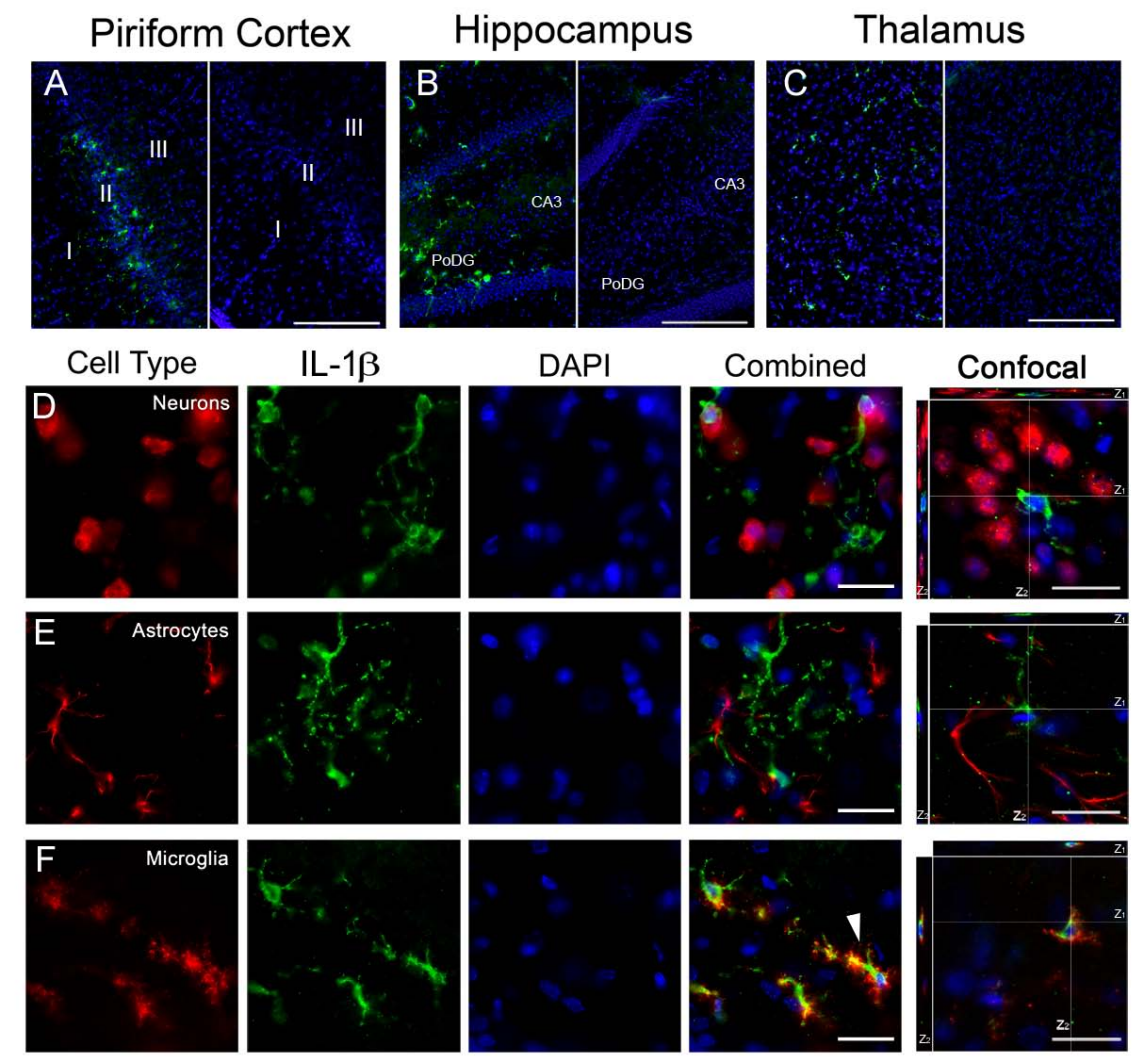
**IL-1β is expressed in activated and dystrophic microglia following GD-induced SE**. IL-1β (green) is present in the piriform cortex, hippocampus and thalamus 12 hours after GD-induced SE (A, B & C; left), though absent in vehicle controls (A, B & C; right). IL-1β had a similar, though less robust, expression pattern compared to IL-1α. IL-1β did not co-localize with either neurons (D, red) or astrocytes (E, red). Co-localization was observed in microglia (F, red), both activated and dystrophic (white arrow). DAPI (A-F, blue) labels nuclei. For confocal images, the white lines indicate the vertical (Z) sectioning of the image and are shown as Z_1 _and Z_2 _on the edges of the main image. (Scale bar: 250 μm (A-C), 50 μm and 10 μm (D-F) for regular and confocal fluorescent microscopy respectively; n = 3 for each cytokine/cell type combination.)

The cytokines IL-1α (Figure 5) and β (Figure 6) (collectively IL-1) are functionally similar and likewise have similar labeling patterns in the piriform cortex (Figure 5A and 6A, left), hippocampus (dentate gyrus shown; Figure 5B and 6B, left) and thalamus (lateral posterior region shown; Figure 5C and 6C, left). Labeling was absent in vehicle (Figure 5 and 6A, B, &6C right) and secondary controls (not shown). In the piriform cortex, IL-1 positive cells were located in all 3 layers with IL-1α found predominantly in layer I and IL-1β found in layer II. In the hippocampus, IL-1 positive cells were found primarily in the polymorphic layer of the dentate gyrus (PoDG) and the CA3 pyramidal layer closest to the dentate gyrus. IL-1 positive cells were also found in the laterodorsal and lateral posterior nuclei of the thalamus. To identify these cells, sections were co-labeled with antibodies specific for neurons, astrocytes or microglia and for IL-1α or β. Positive immunoreactivity for IL-1 was absent in both neurons (Figure 5 and 6D) and astrocytes (Figure 5 and 6E). However, strong and abundant expression of IL-1α and β was observed in many activated microglia (Figure 5F). IL-1β, but not IL-1α, also appeared in morphologically identified dystrophic microglia (Figure 6F, white arrow). Cellular expression of IL-1 was the same regardless of the brain region investigated.

### IL-6 is expressed by neurons and astrocytes following GD induced SE

**Figure 7:**
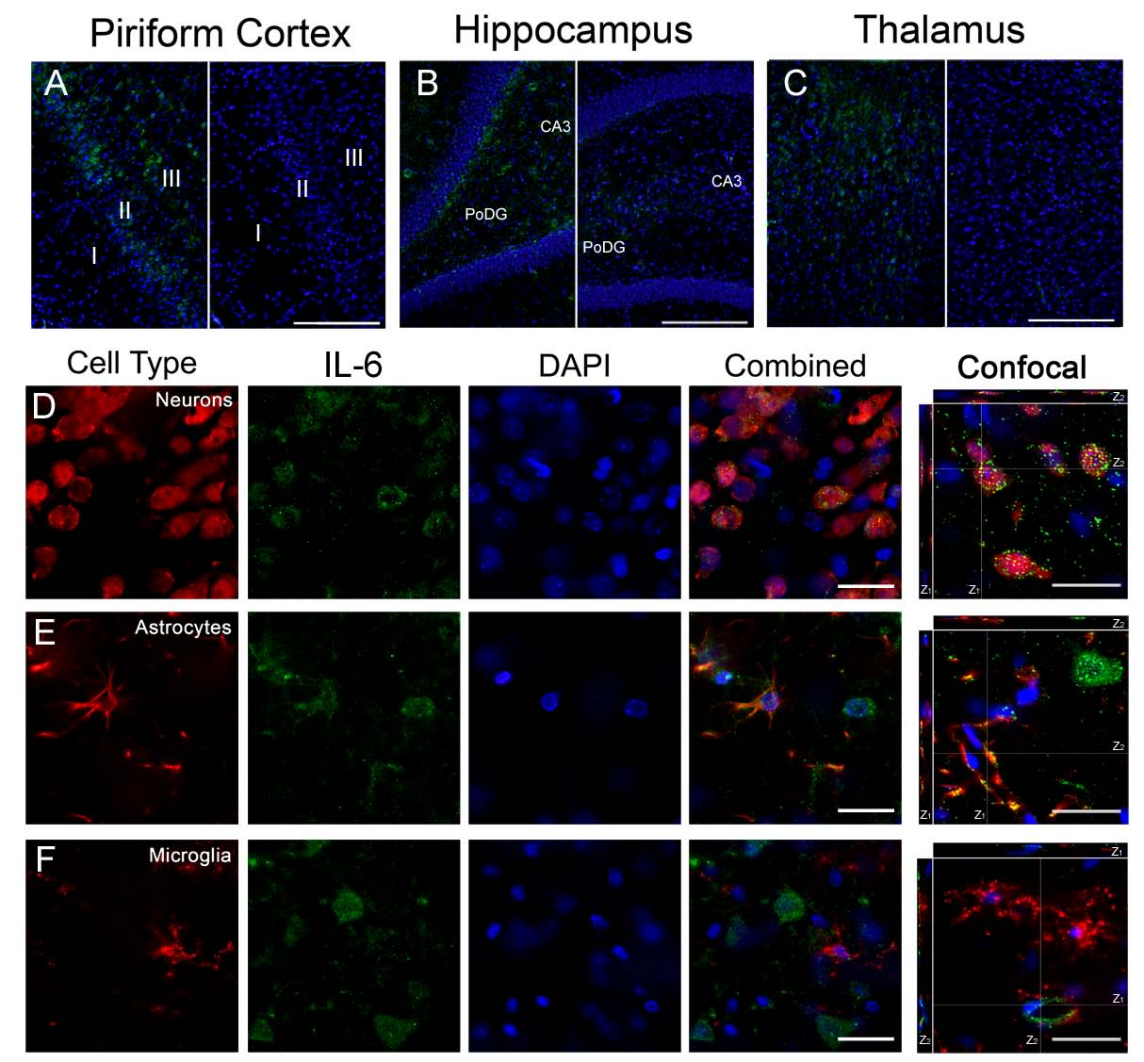
**IL-6 is expressed in neurons and astrocytes following GD-induced SE**. IL-6 (green) is present in the piriform cortex, hippocampus and thalamus 12 hours after GD-induced SE (A, B & C; left), though absent in vehicle controls (A, B & C; right). IL-6 co-localized with neurons (D, red) and to a lesser extent, astrocytes (E, red). No co-localization was observed in microglia (F, red). DAPI (A-F, blue) labels nuclei. For confocal images, the white lines indicate the vertical (Z) sectioning of the image and are shown as Z_1 _and Z_2 _on the edges of the main image. (Scale bar: 250 μm (A-C), 50 μm and 10 μm (D-F) for regular and confocal fluorescent microscopy respectively; n = 3 for each cytokine/cell type combination.)

IL-6 immunolabeling was present in the piriform cortex (Figure 7A, left), hippocampus (dentate gyrus shown; Figure 7B, left) and thalamus (Figure 7C, left). IL-6 labeling was mostly absent in vehicle controls (Figure 7A, B, &7C right), though light diffuse immunoreactivity in a limited number of piriform cortex and thalamus neurons was occasionally observed. Cellular expression of IL-6 was the same regardless of the brain region investigated. IL-6 labeling was moderate to strong, with both a diffuse and punctate distribution in neuronal cell bodies (Figure 7D). IL-6 positive neurons were localized primarily in layers II and III of the piriform cortex, the pyramidal and extrapyramidal regions of the hippocampus and many of the thalamic nuclei. Diffuse labeling of IL-6 was also present in activated astrocytes (Figure 7E) but often sparse and less intense than in neurons. Though co-localization was infrequent, IL-6 was often found coincident with astrocytes around the vasculature in all studied brain regions. No co-localization was observed between IL-6 and microglia (Figure 7F). To confirm that the cellular origin of IL-6 did not change with time, 24 hour tissues were also labeled with IL-6. No differences in cellular expression of this cytokine were observed between 12 and 24 hours (data not shown).

## Discussion

Brain damage caused by CWNA induced seizure activity can cause profound behavioral changes in animals [26,27] and may lead to behavioral impairment and a reduced quality of life for CWNA exposure survivors [28]. Neuroinflammation is common following many types of brain injury, including seizure activity, and may exacerbate brain pathology following GD-induced SE. Our current understanding of the neuroinflammatory process following GD exposure is limited to mRNA transcript [7-10] and protein levels of a small number of factors [23]. Neuroinflammation has been associated with brain pathology in many CNS injury models [29-31] since many inflammatory mediators are toxic to neural cells [16,20]. This study reveals a strong induction of innate inflammatory cytokines in brain regions vulnerable to GD-induced SE.

Here, the regional and temporal protein concentration changes of 10 cytokines were quantified following GD-induced SE. We focused on three brain regions where damage is robust following GD exposure, the piriform cortex, hippocampus and thalamus [6,32]. The protein concentrations of four APR cytokines (IL-1α, IL-1β, IL-6 and TNF-α) significantly increase and compare favorably with previously reported mRNA data [7,9,10]. Transcript work in a mouse model of GD exposure [7] revealed results similar to those reported here for IL-1β and IL-6, though TNF-α protein peaks in the rat model precede their observed mRNA peaks in the mouse model by approximately 12 hours. These discrepancies may be due to differences in HI-6 pretreatment time (5 vs. 30 minutes prior), HI-6 concentration (50 vs. 125 mg/kg), AMN treatment (none vs. 1 minute) or species physiology (mouse vs. rat). The closest comparison to this study reported mRNA expression peaks in the piriform cortex at 2 hours for TNF-α and 6 hours for IL-1β and IL-6, between 10-18 hours before the protein peaks shown in this study [10]. In this case, transcription of the mRNA predictably precedes translation of the protein. However, they saw no significant increases in TNF-α or IL-6 mRNA but did report a significant increase in IL-1β mRNA in the hippocampus, contrary to the protein data reported here. It is unknown why these differences occurred. However, a strong increasing trend is apparent in both the mRNA (TNF-α and IL-6) and protein data (IL-1β) that is consistent with the aforementioned transcription/translation pattern and could conceivably be resolved by more data points.

Neurons, activated microglia, and astrocytes can produce neurotoxic cytokines in the brain following CNS injury [33]. In this study, activated microglia produce both IL-1α and β, whereas dystrophic microglia express only IL-1β. Morphologically, dystrophic microglia display characteristics that may include deramification, spheroid formation, beading along the processes and cytorrhexis [34]. It is well established that IL-1 expression by activated microglia can lead to tissue injury following CNS damage [35,36], but little is known about dystrophic microglia or why they might preferentially express one IL-1 isoform over the other. Dystrophic microglia only appear in progressive neurodegenerative disease states [34,37] and may be indicative of the rapid progression of an acute pathological process in this model. Coupled with the observation that IL-1β is the major form of IL-1 that contributes to CNS damage following injury [38,39], IL-1β expression by dystrophic microglia may represent yet another signal by an injured cell to propagate the inflammatory cascade. Though unable to localize TNF-α to specific cells, expression of this pro-inflammatory cytokine can also be detrimental to neural cells in the injury area. TNF-α does not appear to be directly neurotoxic to neurons, but expression as part of neuroinflammation greatly exacerbates excitotoxic cell death in the presence of excess glutamate [40], a condition that likely occurs following GD exposure [24]. Overall, expression of TNF-α, IL-1α and IL-1β is neurotoxic, contributing to neuronal cell death, edema and blood brain barrier failure following CNS injury [18,41,42] and likely has a similar role following GD-induced SE.

IL-6 was expressed primarily by neurons and, to a lesser extent, by hypertrophic astrocytes in this model. IL-6 expression occurs in both cell types [43-45] and can be neuroprotective *in vivo *[45,46] and *in vitro *[43]. Since neurons are particularly vulnerable to GD-induced SE damage [6,47], expression of a factor that promotes neuronal survival by inducing the expression of metallothionein I + II and granulocyte-macrophage colony-stimulating factor in macrophages [48], stimulates the release of nerve growth factor from astrocytes [49] and inhibits neutrophil infiltration [32] to counter neurotoxic inflammatory factors such as IL-1is not surprising. Furthermore, IL-6 expression begins the synthesis of corticotrophin and glucocorticoids [50], initiating an anti-inflammatory feedback loop. Expression of IL-6 predictably follows early expression of IL-1α, IL-1β and TNFα. Therefore, IL-6 expression by neurons and astrocytes may be a neuroprotective mechanism initiated following the neurotoxic expression of IL-1 in microglia.

## Conclusion

This is the first study to show concurrent upregulation and cellular origin of APR cytokines in the piriform cortex, hippocampus and thalamus following GD-induced seizure activity. The strong induction of IL-1α, IL-1β and TNF-α suggests that a neurotoxic environment is created in the brain in response to seizure activity may be countered by IL-6 expression.

## List of abbreviations

GD: soman; SE: status epilepticus; APR: acute phase response; IL: interleukin; TNF: tumor necrosis factor; CWNA: chemical warfare nerve agent; CNS: central nervous system; MinDC: minimum detectible concentration; IHC: immunohistochemistry; PoDG: polymorphic layer of the dentate gyrus.

## Competing interests

The authors declare that they have no competing interests.

## Authors' contributions

EAJ analyzed all data, wrote the manuscript and participated in acquisition of data. EAJ and RKK both participated in developing the study concept and experimental design. Both authors read and approved the final manuscript.
